# 塞利尼索联合皮下注射地西他滨治疗既往维奈克拉治疗失败的髓系恶性肿瘤疗效分析

**DOI:** 10.3760/cma.j.cn121090-20240920-00358

**Published:** 2025-05

**Authors:** 瑞华 米, 琳 王, 宁 胡, 超 李, 琳 陈, 轶轩 马, 旭东 魏

**Affiliations:** 1 郑州大学附属肿瘤医院/河南省肿瘤医院，郑州 450000 The Affiliated Cancer Hospital of Zhengzhou University/Henan Cancer Hospital, Zhengzhou 450008, China; 2 河南大学淮河医院，开封 475000 Huaihe Hospital of Henan University, Kaifeng 475000, China; 3 南阳市中心医院，南阳 473001 Nanyang Central Hospital, Nanyang 473001, China

## Abstract

维奈克拉（Ven）目前在临床应用的范围越来越广，不管是急性髓系白血病（AML）还是高危骨髓增生异常综合征（MDS），既往Ven治疗后失败的患者，再诱导方案的选择尚无统一标准。本文对10例既往Ven治疗失败的AML和伴原始细胞增多1/2型MDS患者，采用塞利尼索（Selinxor）联合皮下注射地西他滨进行治疗，对其疗效和安全性进行回顾性总结，并结合文献复习。7例AML患者中，完全缓解（CR）1例，CR伴不完全血液学恢复2例，部分缓解1例，未缓解2例，疾病进展1例；3例MDS患者中，骨髓完全缓解2例，疾病稳定1例。6例有效患者的中位缓解持续时间为2（0.5～6）个月。10例患者均出现不同程度的骨髓抑制，5例患者出现轻微胃肠道反应，但均在可控的范围内，整体耐受性良好，没有患者因治疗不良反应而死亡。表明塞利尼索联合皮下注射地西他滨为Ven治疗失败的患者提供了一种新的治疗手段，且耐受性良好。

## Abstract (en)

Venetoclax（Ven）is now widely used for both acute myeloid leukaemia（AML）and high-risk myelodysplastic syndrome（MDS）, yet there is no consensus on salvage regimens after Ven failure. This study retrospectively evaluated the efficacy and safety of selinexor combined with subcutaneous decitabine（DAC）in 10 patients with AML or MDS with excess blasts（MDS-EB1/2）who had experienced prior Ven treatment failure. A literature review was also performed. Among the 7 patients with AML, 1 achieved complete remission（CR）, 2 achieved CR with incomplete hematologic recovery（CRi）, 1 achieved partial remission（PR）, 2 had no remission, and 1 experienced disease progression（PD）. Among the 3 patients with MDS, 2 achieved marrow CR and 1 had stable disease（SD）. The median duration of response among the 6 responding patients was 2 months（range, 0.5–6 months）. All 10 patients experienced varying degrees of myelosuppression. Five patients had mild gastrointestinal reactions, all of which were manageable. The overall tolerability was good, and no treatment-related deaths occurred. These findings suggest that selinexor combined with subcutaneous decitabine offers a novel and well-tolerated therapeutic option for patients with myeloid malignancies who have previously failed venetoclax-based therapy.

维奈克拉（Ven）联合去甲基化药物（HMA）已成为75岁及以上、不适合强化疗的初治急性髓系白血病（AML）患者的治疗新选择。对于难治/复发性（R/R）AML患者，联合用药的总有效率（ORR）达47.9％，中位总生存（OS）期为9.6个月[1]。然而，临床中仍有1/3左右的R/R AML患者对该方案原发耐药，且一线HMA治疗失败的患者，经挽救治疗后疗效很差，中位OS期仅2.9个月[2]。

异基因造血干细胞移植（allo-HSCT）为较高危骨髓增生异常综合征（HR-MDS）的唯一治愈手段，但由于高龄、合并症、供者、费用和移植风险等因素，仅有少数患者可接受allo-HSCT。高清妍等[3]应用Ven联合HMA治疗HR-MDS，ORR为62.5％。

不管是AML还是HR-MDS，既往Ven治疗失败的患者，再诱导方案的选择尚无统一标准。张剑等[4]应用塞利尼索（Selinxor）联合HMA治疗Ven暴露R/R AML患者12例，ORR为66.7％。现回顾性分析郑州大学附属肿瘤医院、河南大学淮河医院及南阳市中心医院应用塞利尼索联合皮下注射地西他滨（DAC）治疗既往Ven治疗失败的10例AML和MDS患者，探讨其疗效和安全性。

## 病例与方法

1. 病例来源：纳入2020年7月至2024年8月在郑州大学附属肿瘤医院、河南大学淮河医院及南阳市中心医院血液科既往接受过Ven治疗的10例AML和伴原始细胞增多1/2型MDS（MDS-EB1/2）患者。10例患者均行骨髓细胞形态学、流式细胞术免疫分型、细胞遗传学、融合基因和基因突变组套等（MICM）分型检查。初治AML诊断及预后分层参照2023年成人AML白血病中国诊疗指南[5]；MDS诊断及预后分层参照2022年WHO第5版造血与淋巴组织肿瘤分类（WHO-HAEM5）[6]。R/R AML的诊断标准参照《中国复发难治性急性髓系白血病诊疗指南（2023年版）》[7]。

2. 治疗方案：塞利尼索+小剂量皮下注射DAC方案具体为：塞利尼索40 mg，每周两次，口服，连用4周（MDS为2～3周）；DAC 10 mg/次，每周3次，皮下注射，连用4周（MDS为2～3周）。治疗期间，依据患者临床特征给予抗感染、成分血输注、重组人G-CSF、止吐等支持治疗。有移植条件的患者行allo-HSCT。

3. 疗效评估：AML疗效评估参照2023年成人AML中国诊疗指南，ORR为完全缓解（CR）率、CR伴部分血液学恢复（CRh）率、完全缓解伴不完全血液学恢复（CRi）率、骨髓无白血病状态（MLFS）率、部分缓解（PR）率之和，复合CR（CRc）率为CR率、CRh率、CRi率之和。

MDS疗效评估参照2022年WHO-HAEM5，分为CR、PR、骨髓完全缓解（mCR）率、血液学改善（HI）、疾病稳定（SD）和治疗失败。ORR为CR率、mCR率、PR率之和。

4. 安全性评估：根据《常见不良反应事件评价标准（Common Terminology Criteria for Adverse Events, CTCAE）》5.0版[8]，对患者用药后发生的不良事件（AE）进行分级。

5. 随访：采用电话、门诊随诊、医院病历登记系统等方式进行随访。随访截至2024年8月31日。缓解持续时间（Duration of response, DOR）是指患者接受含塞利尼索的方案行第1次疗效评估达到CR/CRi/CRh/MLFS/PR至第1次评估为疾病复发或任何原因引起死亡的时间。OS时间指患者接受含塞利尼索的方案治疗开始至随访截止的时间，死亡患者则计算至死亡日。

## 结果

1. 临床特征：10例既往Ven治疗失败的R/R AML和HR-MDS的临床资料见表1。其中AML患者7例，MDS患者3例。男6例，女4例。中位年龄67（29～74岁）。应用塞利尼索方案前患者的中位WBC为2.89（1.06～11.11）×10^9^/L，骨髓原始细胞中位数为25.3％（2.8％～90.5％），异常基因突变的检出频次为RUNX1、DNMT3A、TET2、TP53各3次，BCOR、NRAS、KRAS、ASXL1各2次，PTPN11、FLT-ITD、FLT3-KTD、WT1、NQO1、IDH2、PHF6、SRSF2各1次。

**表1:** 塞利尼索联合小剂量皮下注射地西他滨治疗既往维奈克拉治疗失败的AML和MDS患者的临床特征

例号	性别	年龄（岁）	诊断	细胞遗传学	分子生物学	预后分层	既往治疗方案	复发或难治	WBC（×10^9^/L）	骨髓原始细胞（％）	流式细胞术检测的MRD
1	女	62	AML-M_5_	46,XX[20]	PTPN11、RUNX1、BCOR、OAZ1::E2A融合基因	不良	VA×8; IFNα1b+IL-2+Thal	难治	2.28	5.6	异常髓系原始细胞占0.36％，幼稚及偏成熟单核细胞占4.55％
2	男	70	AML-M_2_	46,XY[20]	FLT3-TKD（VAF: 4.35％）、FLT3-ITD（VAF: 3.9％）、NRAS、DNMT3A、WT1	中等	VA×6;Ven+DAC+Gilteritinib×2; IFNα1b+IL-2+Len+Sorafenib; 临床试验	难治	11.11	90.5	异常髓系原始细胞占71.75％
3	男	68	AML-M_4_	47,XY,+21[10]	DNMT3A、KRAS、NQO1、RUNX1、TET2	不良	VA+Ara-C×2; VA; VA+HHT+Chid; Gilteritinib; AZA+CAG	难治	9.21	48.2	异常髓系原始细胞占10.11％，幼稚单核细胞占40.92％
4	男	74	AML-M_2_	46,XY[20]	TET2	中等	IA×2; MA; HA×3; FLAG; DAC; Ara-C+Vp16; Ara-C×2; VA×3	难治	3.36	43.0	异常髓系原始细胞占22.5％
5	女	64	AML-M_5_	未见分裂象	DNMT3A、IDH2、BCOR	不良	VA×10	复发	1.10	2.8	异常髓系原始细胞占1.16％
6	女	29	AML-M_2b_	47,XX,t（8;21）（q22;q22）, del（9）（q22q34）, +21[20]	KARS、NRAS、ASXL1	良好	DAC+IA; IA×2; ID-Ara-C×2; DAC+Ven×5; VA×3; AZA+CHG+Ven×4; IFNα1b +IL-2+Len	难治	4.07	50.4	异常髓系原始细胞占24.44％
7	男	71	AML-M_5_	44, XY, +Y,?6, +8,−13,−15,−17,−21[20]	TP53（VAF: 44.38％）	不良	VA×2	难治	1.06	32.5	异常髓系原始细胞占7.16％，幼稚单核细胞占21.90％
8	男	65	MDS-EB2	46, XY[20]	ASXL1、PHF6、RUNX1、SRSF2、TET2	极高危	VA; Ven+DAC×6	复发	6.00	18.0	异常髓系原始细胞占16.59％
9	男	73	MDS-EB1	复杂核型	TP53（VAF:65.83％）	极高危	VA×6	复发	2.41	5.4	异常髓系原始细胞占3.42％
10	女	66	MDS-EB1	复杂核型	MLL-PTD（+）、TP53（VAF: 63.67％）	极高危	VA×11	复发	1.35	8.6	异常髓系原始细胞占2.79％

**注** AML：急性髓系白血病；MDS：骨髓增生异常综合征；EB1：伴原始细胞增多1型；EB2：伴原始细胞增多2型；VAF：变异等位基因频率；VA：维奈克拉+阿扎胞苷；IFNα1b+IL-2+Thal：干扰素α1b+白细胞介素-2+沙利度胺；Ven：维奈克拉；DAC：地西他滨；Gilteritinib：吉瑞替尼；Len：来那度胺；Sorafenib：索拉非尼；Ara-C：阿糖胞苷；HHT：高三尖杉酯碱；Chid：西达苯胺；CAG：阿柔比星+阿糖胞苷+G-CSF；IA；伊达比星+阿糖胞苷；MA：米托蒽醌+阿糖胞苷；HA：高三尖杉酯碱+阿糖胞苷；FLAG：氟达拉滨+阿糖胞苷+G-CSF；Vp16：依托泊苷；CHG：高三尖杉酯碱+阿糖胞苷+G-CSF

7例AML患者中，预后良好组1例，预后中等组2例，预后不良组4例；难治性6例，复发性1例。3例MDS患者中，预后分层均为极高危组。10例患者既往均应用HMA药物治疗，其中单独应用阿扎胞苷（AZA）的患者6例，同时应用AZA和DAC的患者4例，无单独应用DAC的患者。

2. 疗效评估：所有患者均可评估疗效。7例AML患者中，CR 1例，CRi 2例，PR 1例，未缓解（NR）2例，疾病进展（PD）1例；3例MDS患者中，mCR 2例，SD 1例。6例有效患者的中位DOR为2（0.5～6）个月。

治疗有效的4例AML患者，均为难治性，且初诊预后分层中预后中等2例，预后不良2例；2例既往单独应用过AZA，2例AZA和DAC均应用过；3例无效AML患者中，2例仅应用AZA，1例AZA和DAC均应用过，且该患者为伴RUNX1∷RUNX1T1融合基因的低危患者。

治疗有效的2例MDS患者，既往仅应用过AZA，且均伴随TP53异常（变异等位基因频率值均大于60％），1例无效的MDS患者，既往应用过AZA和DAC。

塞利尼索方案再诱导治疗有效的患者，继续原方案巩固治疗（本研究入组的塞利尼索治疗有效的患者，因多种原因，无一例行allo-HSCT）；而2例桥接allo-HSCT者，均为塞利尼索方案再诱导失败的患者，截至随访终点，2例患者疾病均处于微小残留病（MRD）阴性CR。所有患者的疗效评估见表2。

**表2:** 塞利尼索联合小剂量皮下注射地西他滨治疗既往维奈克拉治疗失败的急性髓系白血病和骨髓增生异常综合征患者的疗效分析

例号	疗效评估	DOR（月）	OS（月）	是否移植	转归
1	MRD（−）CRi	6	14	否	存活
2	PR	3	5	否	死亡
3	CRi	2	5.5	否	存活
4	CR	1	1.5	否	死亡（脑梗死）
5	PD	0	11	否	存活
6	NR	0	5	UD-HSCT	存活
7	NR	0	3	否	死亡
8	SD	1.5	25	UD-HSCT	存活
9	mCR&HI	2	5	否	死亡
10	mCR	0.5	1.5	否	存活

**注** DOR：缓解持续时间；OS：总生存；MRD：微小残留病；CR：完全缓解；CRi：CR伴不完全血液学恢复；PR：部分缓解；PD：疾病进展；NR：不缓解；mCR&HI：骨髓CR伴血液学改善；SD：疾病稳定；UD-HSCT：无关全相合造血干细胞移植

3. Ven治疗情况分析：7例AML患者中，接受Ven治疗的中位周期为6（2～11）个，塞利尼索方案治疗有效的4例患者中，3例为既往接受过Ven治疗达CR后在巩固治疗期间近期复发，1例为常规化疗中多次复发，Ven挽救治疗达CR后再次复发患者。余3例无效的患者，分别为既往接受过Ven治疗达CR后在巩固治疗期间远期复发1例、化疗联合Ven治疗期间多次复发1例、Ven治疗2周期NR 1例。

3例MDS患者，接受Ven治疗的中位周期为7（6～11）个，均为Ven方案维持治疗期间复发。

4. 不良反应：治疗期间，10例患者均出现不同程度的骨髓抑制。7例患者出现3级或4级的中性粒细胞减少，10例患者均出现3级或4级的血小板减少（其中3例患者治疗前已存在4级血小板减少，且血小板输注无效）。1例患者治疗期间出现粒细胞缺乏伴发热；5例患者出现轻微胃肠道反应（塞利尼索用药当天常规三联抗止吐治疗），但不影响药物持续治疗；2例接受allo-HSCT患者，无急性移植物抗宿主病（aGVHD）发生，移植过程顺利；10例患者均未出现肝肾功能异常。

5. 生存情况：截至随访终点，10例患者中，6例存活（包括2例接受移植的患者），4例死亡，其中3例死于疾病进展，1例死于合并症；中位OS期为5（1.5～25）个月。4例没有接受移植尚生存的患者，均再次进展，其中1例接受中药治疗，1例成分血输注对症处理，2例参加新药临床研究。

## 讨论

Ven目前已在初治不适合强化疗[9]、R/R[1]以及新诊断的适合强化疗[10]的AML患者中广泛应用。然而，Ven治疗失败的患者，预后非常差，其生存期不足3个月[2]。不管是Ven的原发性耐药或适应性耐药，其机制非常复杂，研究表明与多种因素有关，包括FLT3-ITD、RAS/MAPK突变和TP53突变/扩增、烟酰胺代谢升高、c-myc、MCL1高表达等[11]。对于Ven治疗失败后的挽救治疗，迫切需要有无交叉耐药的且不同作用机制的新药组成新的治疗方案。

研究表明，核输出蛋白1（XPO1）的过度表达可能是AML的一个不利预后因素，XPO1表达的升高与AML患者的OS期成反比[12]。塞利尼索是口服的XPO1抑制剂，抑制XPO1能够阻止真核起始因子4E（eIF4E）介导的BCL2和MCL1 mRNA出核翻译，从而同时下调BCL2和MCL1蛋白表达，诱导AML细胞凋亡，与Ven联合可协同增强抗白血病活性[13]。基于以上研究，塞利尼索在一定程度上能够克服Ven耐药。

真实世界中，HMA药物已经成为MDS或AML患者的基石性治疗药物，尤其对老年患者，AZA的应用更加普遍。一项Ⅱ期临床研究揭示对HMA耐药的MDS或AML患者，仍可从塞利尼索中获益[14]。

Ranganathan等[15]报道DAC可以增强塞利尼索的体外抗白血病作用，这些效应可能是通过DNA甲基化被表观遗传沉默的肿瘤抑制蛋白（CDKN1A和FOXO3A）亚群的重新表达介导的，同时由于这些肿瘤抑制蛋白的细胞质-核迁移受XPO1的调控，增强了这些肿瘤抑制蛋白的抗肿瘤效应。因此，塞利尼索与DAC联合应用具有协同的作用。但DAC其体外作用机制与剂量有关：高剂量时可终止DNA复制，低剂量时可抑制DNA甲基转移酶。2015年Saunthararajah等[16]等应用小剂量皮下注射DAC治疗MDS患者，既达到了去甲基化作用，又降低了毒性反应发生。

本研究中，我们采用塞利尼索联合皮下注射DAC的方案治疗既往Ven治疗失败的10例AML或MDS患者，总ORR为60％（6/10），中位OS期为5（1.5～25）个月；6例有效患者的中位DOR为2（0.5～6）个月。很遗憾的是本中心入组的塞利尼索治疗有效的患者，因多种原因，无一例行allo-HSCT，而接受allo-HSCT的2例患者，均为塞利尼索方案再诱导失败的患者，目前均处于无病生存状态。另外，本研究入组的10例患者既往均应用过HMA药物治疗，再次给予DAC治疗，仍然有效，提示DAC和AZA尚无绝对的交叉耐药。另外，本研究方案应用皮下注射小剂量DAC，为本研究的创新之处。

本研究10例患者中7例伴有高危异常基因，其中伴TP53基因异常的3例患者中2例治疗有效，但因入组总患者数有限，尚无法就特定基因异常与该方案的疗效关系得出明确结论。

塞利尼索联合皮下注射DAC为Ven治疗失败的患者提供了再次缓解的机会，一定程度上改善了Ven治疗失败患者的预后，但持续缓解期有待进一步提高，有移植条件的患者尽量桥接allo-HSCT。但因为本研究为小样本量的回顾性分析，未来需要进一步扩大样本量及多中心协作进一步验证该方案的疗效，甚至与其他方案进行疗效对比。
